# Efficacy of *E. officinalis* on the Cariogenic Properties of *Streptococcus mutans*: A Novel and Alternative Approach to Suppress Quorum-Sensing Mechanism

**DOI:** 10.1371/journal.pone.0040319

**Published:** 2012-07-05

**Authors:** Sadaf Hasan, Mohd Danishuddin, Mohd Adil, Kunal Singh, Praveen K. Verma, Asad U. Khan

**Affiliations:** 1 Medical Microbiology and Molecular Biology Laboratory, Interdisciplinary Biotechnology Unit, Aligarh Muslim University, Aligarh, India; 2 National Institute of Plant Genome Research, Aruna Asaf Ali Marg, New Delhi, India; University of Kansas Medical Center, United States of America

## Abstract

The present study was focused on evaluating the potential of *Emblica officinalis* against cariogenic properties of *Streptococcus mutans*, a causative microorganism for caries. The effect of crude extract and ethanolic fraction from *Emblica officinalis* fruit was analysed against *S. mutans*. The sub-MIC concentrations of crude and ethanolic fraction of *E. officinalis* were evaluated for its cariogenic properties such as acid production, biofilm formation, cell-surface hydrophobicity, glucan production, sucrose-dependent and independent adherence. Its effect on biofilm architecture was also investigated with the help of confocal and scanning electron microscopy (SEM). Moreover, expression of genes involved in biofilm formation was also studied by quantitative RT- PCR. This study showed 50% reduction in adherence at concentrations 156 µg/ and 312.5 µg/ml of crude extract and ethanolic fraction respectively. However, the biofilm was reduced to 50% in the presence of crude extract (39.04 µg/ml) and ethanolic fraction (78.08 µg/ml). Furthermore, effective reduction was observed in the glucan synthesis and cell surface hydrophobicity. The qRT-PCR revealed significant suppression of the genes involved in its virulence. Confocal and scanning electron microscopy clearly depicted the obliteration of biofilm structure with reference to control. Hence, this study reveals the potential of *E. officinalis* fruit extracts as an alternative and complementary medicine for dental caries by inhibiting the virulence factors of *Streptococcus mutans.*

## Introduction

Dental caries is an irreversible localized infection that results in the progressive tooth decay [1]. *Streptococcus mutans*, a gram positive oral bacterium has been long implicated to be a primary causative agent of this ubiquitous disease [2]. This bacterium is also known to cause infective endocarditis which may cause significant morbidity and mortality [3]. Oral streptococci can enter the bloodstream via bruises in oral cavity and attach to platelet-fibrin-matrices, formed on injured endothelial tissue. The adherence of *S. mutans* to damaged heart tissue is an important event in the pathogenesis of chronic infective endocarditis [4]. *S. mutans* has developed several distinctive mechanisms for its unhindered survival in the oral cavity [5]. The key virulence factors include acidogenicity and aciduricity coupled with its ability to produce large amounts of extracellular polysaccharides or glucans which are synthesized from dietary carbohydrates by glucosyltransferases (GTFs). *S. mutans* is known to produce at least three forms of GTFs: GTF B, GTF C and GTF D, synthesizing mostly insoluble glucan, mixture of soluble and insoluble glucan and soluble glucan respectively. These glucans are essential for the structural formation and establishment of cariogenic plaque [6]. The production of acid by *S. mutans* and its power to tolerate the acidic pH favours its continual survival and colonization in the dental biofilm [7]. These acids thus begin the dissociation of the tooth enamel consequently leading to localized decalcification, cavity formation and breakdown of calcified dental tissue [8]. Thus an important approach for the prevention of its survival in the oral cavity (leading to dental caries) is the inhibition of GTFs. This has been the aim of many *in vitro* studies where different agents including plant extracts and other natural products shown to have these inhibitory features [9].

Recently, there is a worldwide renewal of interest in “green medicine” and an increasing demand for more drugs from plants sources. Thus, natural products continue to be an effective source of several bioactive compounds with no side effects [10]. *Phyllanthus emblica* (syn. *Emblica officinalis*), belongs to the family Phyllanthaceae. It is commonly known as the Indian gooseberry (or amla). It is one of the most celebrated herbs in the Indian system of traditional medicine. Many researches demonstrated its wide array of pharmacological activities, such as anti-pyretic and analgesic activity [11], anti-tumor activity and anti-oxidant activity [12].

In spite of the various pharmacological significance, the activity of *Emblica officinalis* against *S. mutans* causing dental caries, has not been studied in great detail. Therefore, the aim of the present study was to understand the mode of action of *E. officinalis* on inhibition of biofilm formation and adherence properties *S. mutans*.

## Materials and Methods

### Ethics Statement

Not applicable since no animal or human subject was used in this study. Only MTCC strain of *S. mutans* was used for in vitro study.

### Plant Material

Fruits of *Emblica officinalis* were purchased from the local market of Aligarh and the species was confirmed and validated at department of Botany, A.M.U., Aligarh, India.

### Extraction and Preparation of the Herbal Extract

The fruits were washed with distilled water. This was done to reduce the microbial load of the plant material. They were allowed to air dry and were grinded to powder form. The fruit powder was exhaustively extracted using different solvent of increasing polarities (60–80°C) using soxhlet apparatus. The extract was then filtered using Whatman no. 2 filter paper (Whatman International Limited, Kent, England) and filtrate thus obtained was concentrated using vacuum evaporator. Solvents used were of Laboratory Reagent (LR) grade.

### Bacterial Strain

The bacterial strain that was used in this study was *S. mutans* MTCC 497 (Institute of Microbial Technology, Chandigarh, India), which was grown in Brain Heart Infusion (BHI) Broth (Himedia Labs, Mumbai, India) at 37°C.

### GC-MS of *Emblica Officinalis* Crude and Ethanolic Fraction

Constituents of crude extract and ethanolic fraction were analyzed by GCMS-QP2010 plus, which have Rtx® gas chromatograph column (SLB60 m×ID0.25 mm × thickness 0.25 µm). Column oven temperature was set at 80°C for 1 min, which was increased to 180°C at the rate of 10°C min^−1^ with 4 min hold and finally increased to 300°C at the rate of 15°C min^−1^ with 17 min hold. The injection temperature was 270°C. The flow control mode was set at linear velocity while the ionization voltage mode was 70 eV. Helium was employed as a carrier gas with a constant flow at 1.21 ml min^−1^. The major constituents of the crude and ethanolic fractions were identified by drawing a comparison between the GC retention time with MS reference database of NIST05s.LiB. The major constituent of each extract was purchased from Sigma Aldrich, U.S.A. The MIC and other biochemical assays were performed using these compounds.

### Determination of Bacteriostatic (MIC) and Bacteriocidal (MBC) Concentration

The MIC and MBC of the extracts against *S. mutans* was determined by microdilution method [13]. Briefly, *S. mutans* was treated with increasing concentrations of the extracts concentrations ranging from 2.44 to 5000 µg ml^−1^ in a series of two fold dilutions. The MIC was determined as the lowest concentration that totally inhibits visible bacterial growth. MBC, on the other hand was determined by subculturing the test dilutions on a tryptic soya agar plates and incubated further for 24 h. All these determinations represent the mean of three independent experiments.

### Effect on Sucrose-dependent and Sucrose-independent Adherence to Smooth Glass Surfaces

The effect of the sub-MIC concentrations of the extracts on *S. mutans* was studied as inhibition of adhered cells on smooth glass surface [14]. The bacteria were grown at 37°C for 24 h at an angle of 30° in a glass tube containing 10 ml of BHI with or without 5% (w/v) sucrose and sub-MIC concentrations of the extracts. The solvent controls included BHI with (sucrose dependent) and without sucrose (sucrose independent) and equivalent amounts of DMSO and ethanol. After incubation, the glass tubes were slightly rotated and the planktonic cells were decanted. The adhered cells were then removed by adding 0.5 M of sodium hydroxide followed by agitation. The cells were washed and suspended in saline. The adherence was quantified spectrophotometrically at 600 nm. All these determinations were performed in triplicates, using untreated BHI medium as control.

Percentage adherence  =  (O.D. of adhered cells/O.D. of total cells) ×100.

### Collection of Saliva

Under masticatory stimulation by chewing a piece of parafilm, the saliva was collected from a healthy individual who was abstained from tooth brushing and eating for 5 hours prior to collection. The collected saliva was centrifuged at 8000 g for 15 min to obtain clarified saliva and was stored at −80°C [13]. Clarified saliva (100 µl) was added to each well of the plate, where cells have to be inoculated in the presence of saliva. The plate was then incubated at 37°C for 2 h to coat the wells by forming a thin membrane called the salivary pellicle. After incubation, these plates were rinsed thrice with 100 µl PBS before adding the bacterial culture.

### Biofilm Formation Assay

Biofilm formation assay was carried out in flat bottomed 96- wells microtitre plates [15]. The biofilm ability of *S. mutans* was studied in the presence and absence of salivary pellicle. Over-night culture of *S. mutans* was inoculated in fresh BHI medium containing 5% (w/v) sucrose and was grown at 37°C under anaerobic condition to the mid- log phase (1.0 OD_600_). The cultures were then diluted to 1∶100 in pre-warmed media. As described earlier, the wells were first coated with saliva before the addition of bacterial suspensions. The coated plates were again incubated at 37°C for 2 h and were washed thrice with PBS followed by air drying for 30 min. Bacterial suspension (200 µl) was added in the wells of both uncoated and coated plates with varying concentrations of the plant extracts (2.44–5000 µg ml^−1^). Inoculated media treated with cholorohexidine was taken as positive control, inoculated media treated with ethanol as negative control and media alone was taken as blank control. After inoculation, all the plates were incubated for 24 h at 37°C. The culture was then decanted and the plates were lightly washed thrice with 200 µl of sterile distilled water to remove planktonic and other loosely bound cells. The attached bacterial cells were stained with 50 µl of 0.1% crystal violet for 15 min. After rinsing twice with 200 µl of sterile water, the bound dye was removed from the stained cells using 200 µl of 99% ethanol. Plates were then placed on a shaker for 5 min to allow full release of the dye. Biofilm formation was then quantified by measuring optical density of the suspension at 600 nm by a microplate reader (BIO- RAD iMark ^TM^ Microplate reader, India).

### Inhibition of Water-insoluble and Soluble Glucan Synthesis

The crude GTFase were prepared and assayed to evaluate the effect of plant extracts on glucan synthesis [16]. The cell-free enzymes were precipitated from culture supernatant of *S. mutans* by adding solid ammonium sulphate to 70% saturation called as ammonium cut. The mixture was stirred at 4°C for 1 h and allowed to stand for another 1 h in the cold condition. The precipitate was collected by centrifugation at 12000 g at 4°C for 20 min and dissolved in a minimum volume of 20 mM phosphate buffer (pH 6.8) and then dialysed against 2 mM phosphate buffer (pH 6.8) at 4°C for 24 h. The crude enzymatic preparation was stored at −70°C and was further used for synthesis of water-soluble and insoluble glucan. A reaction mixture consisting of 0.25 ml of crude enzyme and varying concentrations of the extracts in 20 mM phosphate buffer (pH 6.8) containing 0.25 ml of 0.4 M sucrose was incubated at 37°C for 18 h. The fluid was removed post incubation and the tube contents were washed with sterile water. Total amounts of water-soluble and insoluble glucan were measured by the phenol–sulphuric acid method [17]. Three replicates were made for each concentration of the extracts.

### Effect on Cell-surface Hydrophobicity of *S. mutans*

The cell surface hydrophobicity of *S. mutans* was measured according to Microbial adhesion test to hydrocarbon [18]. For cell surface hydrophobicity, cells grown in BHI medium supplemented with 5% sucrose with different concentrations of the extracts. These cells were washed twice and suspended in sterile saline (0.85%) so that their optical density (O.D.) was 0.3 at 600 nm. The cell suspension (3.0 ml) was placed in tubes and 0.25 ml of toluene was added. The tubes were agitated uniformly in a vortex mixer for 2 min and allowed to equilibrate at room temperature for 10 min. Followed by toluene phase separation from the aqueous phase, the O.D. of the aqueous phase was determined spectrophotometrically at 600 nm. Controls consisted of cells which were incubated with or without ethanol. *Streptococcus mutans* with a hydrophobic index >70% was arbitrarily classified as hydrophobic.

### Effect on Acid Production

Effect of different concentrations of the plant extracts on acid production of the *S. mutans* was assessed according to earlier methodology [19]. 5 ml of BHI broth containing 5% (w/v) of sucrose and the different concentrations of the extracts (ranging from 39 to 625 µg ml^−1^) were inoculated with 100 µl of 18 h cultures of *S. mutans* to obtain a final inoculum of 1.5×10^4^ CFU per ml and incubated at 37°C for 24 h. The pH of the bacterial broth was assessed at the onset and after 24 h of incubation. All determinations were performed in triplicates using growth controls.

### Effect on Growth

The effect of the sub-MIC concentration of the extracts (CR - 312.5 µg/ml, ETH - 625 µg/ml) was tested on the growth of *S. mutans.* Aliquots of overnight broth culture of *S. mutans* were inoculated into the tubes to obtain a final inoculum of 1.5×10^4^ CFU per ml followed by addition of the extracts and incubated at 37°C. The growth rate was monitored spectrophotometrically (UV mini 1240, UV- VIS Spectrophotometer Shimadzu) by measuring the absorbance of the culture at 600 nm every hour throughout 24 h of incubation. All determinations were performed as triplicates using growth controls.

### Confocal Microscopy

To analyse the effect of the crude extracts on biofilm, cells were grown on saliva-coated glass coverslips. *Streptococcus mutans* was grown in BHI supplemented with 5% sucrose in a 12-well microtitre plate [20]. The experiment was done in triplicates. Sub-MIC concentration of the extracts was taken while the control was untreated. The wells were inoculated and incubated at 37°C for 24 h. The coverslips were removed from the media and gently washed with sterile PBS to remove the unattached cells. Coverslips were then stained with propidium iodide for 1 h. Fluorescence emission was observed using confocal scanning laser microscope (Fluoview FV200). The excitation wavelength was 594 nm. Images were obtained using 60× oil. The images were obtained with PLAPON 60×1.42 objective with an additional zoom of ×3. Each biofilm was scanned at five randomly selected positions. Each stack of an experiment was examined, and the threshold value that fits best for all image stacks of a trial was chosen. The images of the control and images in the presence of extracts were averaged and compared.

### Scanning Electron Microscopy

The effect of the plant extracts on biofilm was also observed by scanning electron microscopy (SEM). The cells were grown on sterile saliva-coated glass coverslips by dipping them in 12-well cell culture plate. The experiment was done in triplicates. Sub-MIC concentration of the extracts was taken while the control was untreated [21]. The wells were inoculated and incubated at 37°C for 24 h. The coverslips were removed after 24 h and washed three times in sterile PBS. The resultant samples were fixed with 2% formaldehyde and 2.5% glutaraldehyde in PBS (pH 7.4) overnight. After fixing samples rinsed three times with PBS, and dehydrated in absolute ethanol series (ethanolic dehydration). Samples were then completely dried, coated with gold, and observed by scanning electron microscope.

### Effect on Surface Protein Ag I/ II

The total cellular protein from *S. mutans* was conjugated to rabbit anti-Ag I/II to compare and calculate the levels of Ag I/II protein. The amount of protein Ag I/II from control and treated samples was calculated. 10 µg of total protein prepared from the treated (with crude and ethanolic fraction) and untreated (control) *S. mutans* was dissolved in 100 µl of 20 mM carbonate buffer (pH 9.3) was coated on the polystyrene plates. The plates were washed with PBS-Tween and then blocking was done with 5% skimmed milk in bicarbonate buffer. The plates were washed with PBS-T thrice and then incubated with rabbit polyclonal Ag I/II antibody for 2 hours at 37°C. The plates were again washed thrice with PBS and the incubated for 2 hours with 100 µl of anti-rabbit peroxidase coated antibody, dilutions ranging from 1∶100 to 1∶1000000. The plates were washed thrice with PBS and the 50 µl of TMB (3, 3′, 5, 5′- tetramethylbenzidine). The reaction was stopped immediately after appearance of color using 50 µl of 4 N H_2_SO_4_.

### Characterization of Binding Sites in Surface Protein Ag I/ II for Different Compounds of the Crude Extract and Ethanolic Fraction

Crystal structure of C-terminal Region of *Streptococcus mutans* Antigen I/II was downloaded from protein databank having PDB ID: 3QE5. To prepare the protein for docking, all waters were removed from the structure. Further hydrogen atom was added to the Antigen I/II protein. Program Q-Site Finder [22] was used for active site detection.

### Docking Analyses

Two dimensional structures of selected compounds were downloaded from Pubchem database. GOLD 5.0 version [23] was used to study the binding orientation of selected compounds into the *Streptococcus mutans* Antigen I/II structure. The default parameters of the automatic settings were used to set the genetic algorithm parameters. Best protein-ligands complexes were selected based on the scoring function of GOLD fitness score.

### RNA Isolation and Real-time RT-PCR

To analyze the effect of the crude and ethanolic fractions on the gene expression, *S. mutans* was cultured in BHI medium supplemented with sub MIC concentration of the extracts which were deficient of any bactericidal activity against the biofilm cell under examination. Bacteria were diluted at a ratio of 1∶50, inoculated into BHI media and grown for overnight at 37°C. RNA was isolated and purified by using Tri-Reagent (Sigma–Aldrich, St. Louis, MO, USA). A reverse transcription (RT) reaction mixture (20 µL) containing 20 ng of random hexamers, 10 mM dNTPs mix and 1 µg of total RNA sample was incubated at 65°C for 5 min to remove any secondary structure, and placed on ice. Then 10× RT buffer, 25 mM MgCl_2_, 0.1 M DTT, 40 U of RNaseOUT Recombinant Ribonuclease Inhibitor and 50 U of Super Script II RT (Invitrogen, Life Technologies, Carlsbad, California, USA) were added to each reaction mix. After incubation at 25°C for 10 min, the mix was incubated at 42°C for 50 min. Heating the mixture at 70°C for 15 min terminated the reaction, and the cDNA samples were stored at −20°C until used.

The gtfC, GbpB, smu630, Com DE and vicR primers were designed using the algorithms provided by Primer Express (Applied Biosystems) for uniformity in size (≈95 bp) and melting temperature. The primer sequences are provided in table 1. PCR cycle included an initial denaturation at 95°C for 10 min, followed by a 40-cycle amplification consisting of denaturation at 95°C for 15 s and annealing and extension at 60°C for 1 min. The expression levels of all the tested genes were normalized using the 16 s rRNA gene of *S. mutans* as an endogenous control.

**Table 1:** Nucleotide sequences of primers used in this study.

	Primer sequence (5′- 3′)	
Gene	Forward	Reverse
***com DE***	ACAATTCCTTGAGTTCCATCCAAG	TGGTCTGCTGCCTGTTGC
***gtf C***	GGTTTAACGTCAAAATTAGCTGTATTAGC	CTCAACCAACCGCCACTGTT
***vic R***	TGACACGATTACAGCCTTTGATG	CGTCTAGTTCTGGTAACATTAAGTCCAATA
***gbp B***	ATGGCGGTTATGGACACGTT	TTTGGCCACCTTGAACACCT
***smu0630***	GTTAGTTCTGGTTTTGACCGCAAT	CCCTCAACAACAACATCAAAGGT

### Statistical Analysis

All the experiments were performed in triplicates. For each result, data was summarized as mean±standard deviation (SD). Statistical analysis was performed using SPSS (version 11.5, Chicago). For quantitative real time PCR, one tail Student’s t-test was used to calculate the significance of the difference between the mean expression of a given experimental samples and the control samples. A p-value of <0.05 was considered significant.

## Results

### GC-MS of *Emblica Officinalis* Crude Extract and Ethanolic Fraction

The major constituents of the crude and ethanolic fractions of *E. officinalis* were determined by GC- MS. The dominant component in case of the crude extract and ethanolic fraction was 1, 2- Benzenedicarboxylic acid or Phthalic acid (48.52%) and 2- furfuraldehyde (26.94%) respectively. Other components are shown in Table 2.

**Table 2:** Major constituents of the extracts determined by GC- MS.

S.no	Compounds in crude extract	Percentage
**1**	1,2- Benzenedicarboxylic acid/ Phthalic acid	48.52
**2**	Heptanediol	17.42
**3**	Stearic acid	9.30
**4**	Diethyl malate	6.11
**5**	5- hydroxymethyl furfural	4.50
**6**	Methyl palmitate/ methyl hexadecanoate	3.54
**7**	Ethyl linoleate	3.36
**8**	Pyrrolidonecarboxylic acid	2.11
**9**	Ethyl stearate	0.83
**10**	Diethyl phthalate	0.73
**11**	Alpha- linolenic acid	0.67
**12**	Glutaconic anhydride	0.64
**13**	Hexadecane	0.47
**14**	2,3- dipropyl- cyclopropanecarboxylic acid	0.29
**15**	Benzocycloheptene	0.23
**16**	2- furfuraldehyde	0.18
**S.no**	**Compounds in ethanolic fraction**	**Percentage**
**1**	5–(hydroxymethyl) furfural/ Furfuraldehyde	26.94
**2**	Diethylhexyl phthalate	17.33
**3**	Diethyl phthalate	13.09
**4**	Diethyl malate	12.42
**5**	Pidolic acid	8.43
**6**	5- Oxotetrahydrofuran -2- carboxylic acid	4.28
**7**	Heptadecane	2.80
**8**	Glutaconic anhydride	2.68
**9**	Hexadecane	2.40
**10**	Furaldehyde	2.27
**11**	Elaidic acid	1.80
**12**	Tetradecane	1.73
**13**	Linoleic acid	1.08
**14**	Octadecanoid acid	1.02
**15**	Ethyl- 3- ethoxypropanoate	0.84
**16**	2,4,6- octatriene	0.49

### Minimum Inhibitory Concentration (MIC) and Minimum Bacteriocidal Concentration (MBC)

The minimum inhibitory concentration (MIC) of the crude and ethanolic fraction against *S. mutans* was found to be 625 µg/ml and 1250 µg/ml respectively. Inoculum from each well with no visible growth was sub-cultured on tryptic soya agar plates. The maximum concentration that completely inhibited the growth on the plate was found to be 1250 µg/ml and 5000 µg/ml for CR and ETH respectively. However, the MIC of the Phthalic acid and Furfuraldehyde was found to be 250 µg/ml and 500 µg/ml respectively.

### Effect on Sucrose-dependent and Independent Glass Surface Adherence

**Figure 1:**
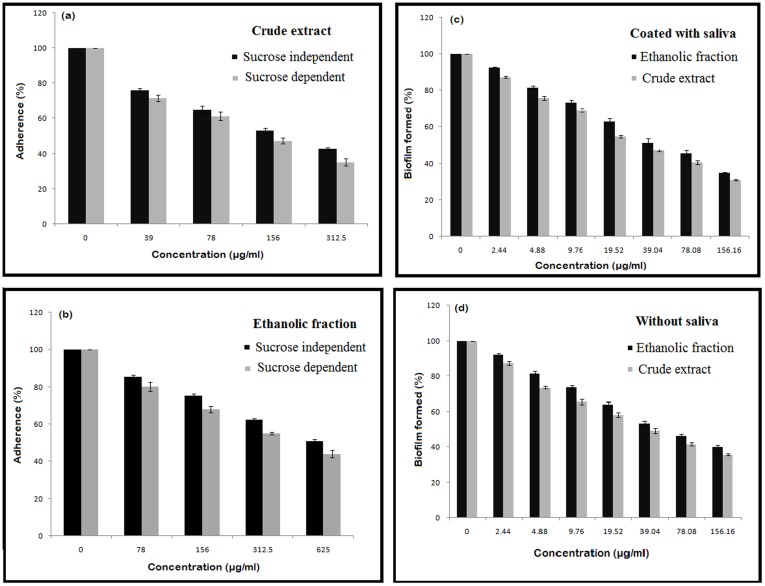
Inhibitory effect of crude and ethanolic fractions on:(**a & b**) **the glass-dependent adherence by** ***S.mutans*** **in the absence (sucrose-independent) and presence of 5% sucrose (sucrose-dependent),** (**c & d**) **Biofilm formation in the presence and absence of salivary pellicle.** Each value is an average of triplicate assays, and each bar indicates±standard deviation (n = 3).

The inhibitory effects of different concentrations of crude and ethanolic fractions on adherence of *S. mutans* to glass tubes are shown in Figure 1 (a & b). The extracts of *E.officinalis* inhibited both sucrose-independent adherence as well as sucrose-dependent adherence. However, the inhibition in sucrose-dependent adherence was more pronounced. Crude extract (156 µg/ml) and ethanolic fraction (625 µg/ml) reduced the adherence of *S. mutans* by 50%. Whereas, Phthalic acid and furfuraldehyde showed only 15–20% reduction in the adherence at sub- inhibitory concentration.

### Biofilm Formation by *S. mutans*

The crude and ethanolic fractions of *E. officinalis* inhibited biofilm formation in a dose-dependent manner as shown in Figure 1 (c & d). The crude extract and ethanolic fraction at a concentration of 39.04 µg/ml reduced the biofilm formation to approximately 50 % in the presence of salivary pellicle. However, in the absence of salivary pellicle, the biofilm reduction was comparatively less affected. Phthalic acid and furfuraldehyde, on the other hand, reduced the biofilm formation to only 15%.

### Inhibition of Water-soluble and Insoluble Glucan Synthesis

**Figure 2:**
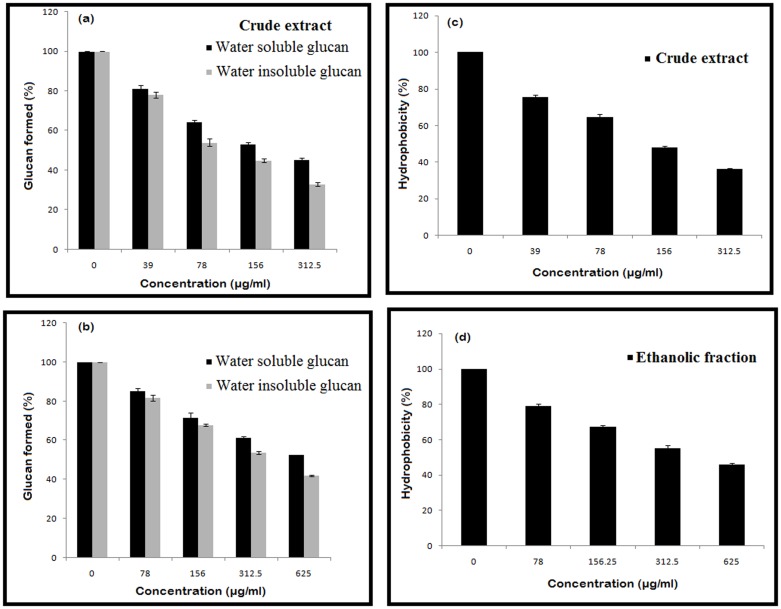
Effect of crude and ethanolic fractions on: (a & b) the inhibition of water- soluble and insoluble glucan synthesis, (c & d) reduction in the hydrophobicity. Each value is an average of triplicate assays, and each bar indicates±standard deviation (n = 3).

The effect of different concentrations of both the extracts (CR and ETH) was assessed for the synthesis of water soluble and insoluble glucan. The synthesis of the glucan was efficiently inhibited in a concentration-dependent manner. The crude extract at a concentration as low as 78 µg/ml and ethanolic fraction at 312.5 µg/ml reduced the formation of water soluble and insoluble glucan to approximately 50% as shown in figure 2 (a & b). However, the reduction was observed to be more significant in case of water-insoluble glucan.

### Effect of the Compound on the Hydrophobicity of *Streptococcus mutans*

It was shown that the cell surface hydrophobicity of *S. mutans* was drastically reduced in a concentration- dependent manner as shown in figure 2 (c & d). The crude extract at a concentration of 156 µg/ml reduced the hydrophobicity to more than 50% and ethanolic fraction reduced the hydrophobicity to approximately 50% at a concentration of 312.5 µg/ml. While the purified compounds did not show any significant reduction in the surface hydrophobicity.

### Effect of Crude and Ethanolic Fractions on the pH of *Streptococcus mutans*

Both the extracts proved to be efficient in reducing the acid production (Table 3). There was a significant change from acidic to alkaline pH with gradual increase in concentration of the extracts. Crude extract and ethanolic fraction at a concentration of 312.5 µg/ml increased the onset acidic pH 4.43 to pH 6.25 and 5.78 respectively. However the active components of these extracts did not show any marked reduction in the acid production.

**Table 3:** Effect of the extracts on the pH of Streptococcus mutans.

Concentration of crude extract (µg/ml)	pH±SD (onset)	pH±SD (after 24 hrs)
**0**	7.48±1.08	4.43±0.05
**39**	7.42±1.13	5.18±1.11
**78**	7.38±0.09	5.42±1.20
**156**	7.41±0.15	5.94±1.60
**312.5**	7.42±1.33	6.25±1.02
**Concentration of ethanolic fraction (µg/ml)**	**pH±SD (onset)**	**pH±SD (after 24 hrs)**
**0**	7.47±0.09	4.43±0.04
**78**	7.40 ±1.13	5.02±0.99
**156**	7.38±0.05	5.31±1.01
**312.5**	7.41±1.02	5.78±0.15
**625**	7.40±0.08	6.08±0.19

Different concentrations of the extracts were added to 1.5×10^4^ CFU ml^−1^ S. *mutans* cells. the pH of was recorded after 24 h of incubation at 37°C. Each concentration was taken in triplicates.

### Effect on Growth

**Figure 3:**
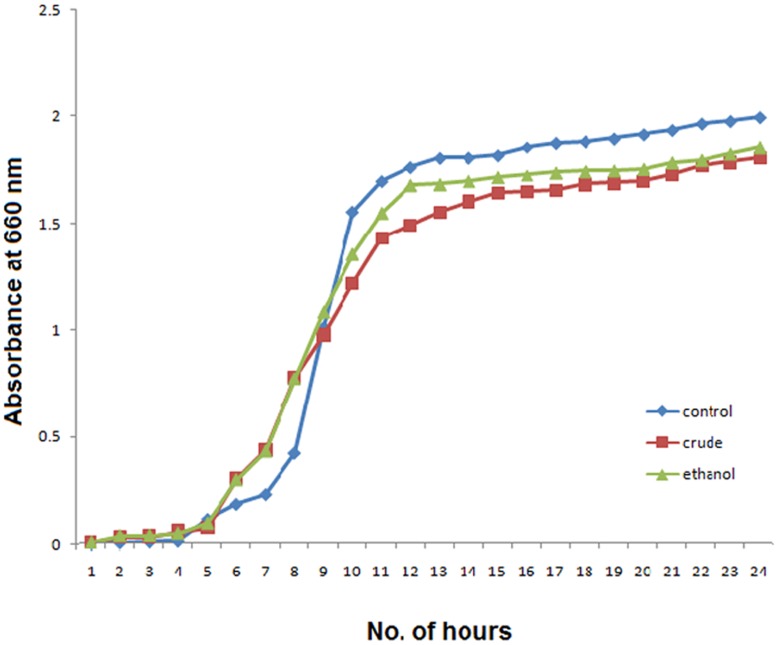
The growth curve of untreated and treated *S.mutans* cells over 24 h.

The effect of the extracts on the growth of *S. mutans* cells is shown in figure 3. There was no significant change in the growth pattern of the control and trtoeated cells.

### Reduction in the Biofilm Architecture Visualized by Microscopy

**Figure 4:**
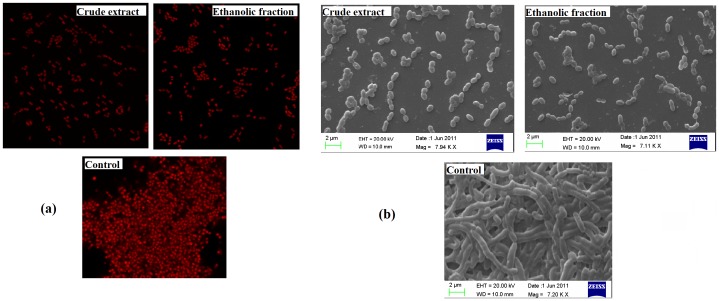
CLSM images (a) and SEM (b) of *Streptococcus mutans* biofilm formed in the presence and absence of the extracts after 24 h of incubation. The assays were performed in triplicates and similar results were obtained.

The structure of the biofilm of *S. mutans* was examined under the confocal laser scanning microscope to observe the changes shown in Figure 4 (a). In the absence of the extracts (control), the cells showed clumping which was not seen in case of samples treated with sub inhibitory concentration of the extracts. Unlike control, the cells in treated samples were clearly dispersed and no clump formation was observed. The Scanning electron microscopy depicted the influence of these plant extracts to reduce the synthesis of extracellular polysaccharides as shown in Figure 4 (b). The control sample clearly showed robust clumping in the exopolysaccharide pool while the cells in the treated sample were evidently dispersed, suggesting the reduction in the formation of extracellular polysaccharides and ultimately reducing the biofilm (plaque) formation.

### Effect on Surface Protein (Ag I/ II)

**Figure 5:**
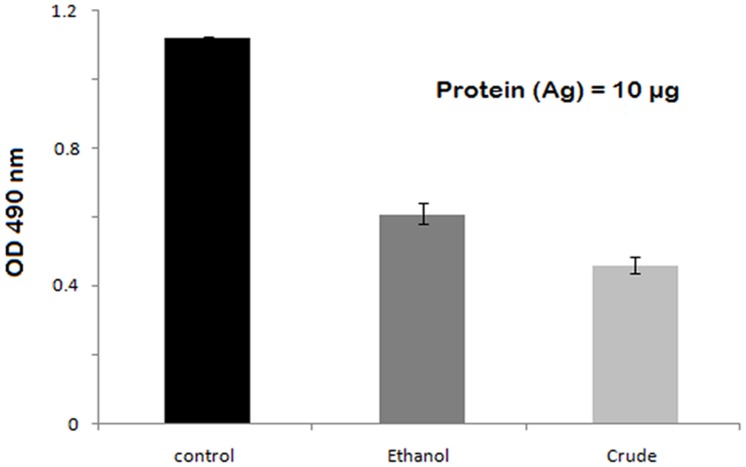
Direct binding ELISA of total protein from untreated *S. mutans* (control) and *S. mutans* treated with crude and ethanolic fraction against polyclonal antibodies of Ag I/II rose in rabbit.

The reduction of protein in samples treated with crude extract was found to be 60% at 1∶100 antibody dilution whereas samples treated with ethanolic fraction showed approximately 50% reduction (Figure 5).

### Docking Studies on Surface Protein Ag I/ II with Different Compounds of Crude Extract and Ethanolic Fraction

**Figure 6:**
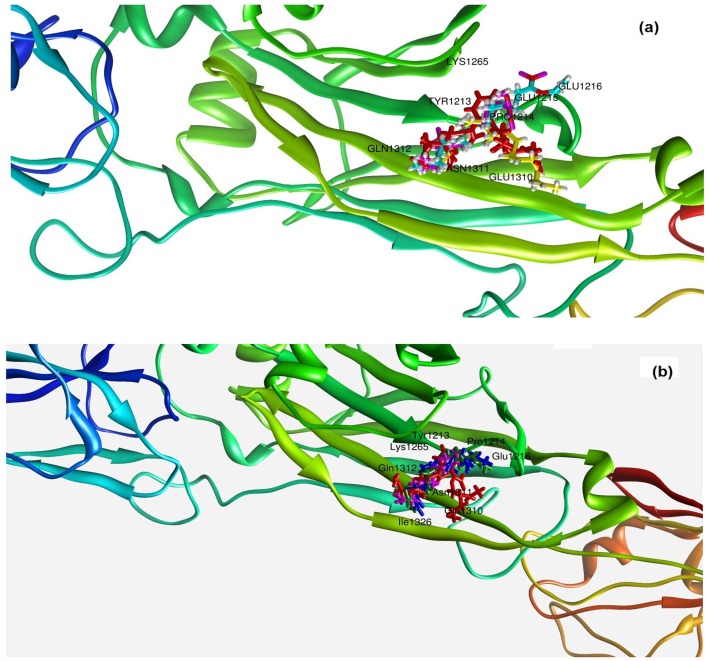
Binding pattern of the compounds analysed by GC-MS showing best docking score from: (a) crude extract and (b) ethanolic extracts within the active site of Ag I/ II.

Four ligands from each; crude extract (Ethyl stearate, phthalic acid, methyl palmitate and ethyl linoleate) and ethanol fraction (hexadecane, elaidic acid, linoleic acid and octadecanoid acid) were identified as the best compounds among all the molecules depicted by GC- MS (Table 2). Compounds from crude extract were found to dock in the active site of target protein with a range of 80–86 Gold fitness score. Whereas compounds from ethanol extract were found to interact with a range of 76–82 Gold fitness score. Eight amino acids (Tyr1213, Pro1214, Glu1215, Glu1216, Lys1265, Glu1310, Asn1311 and GLn1312) were found to be common for all the compounds in stabilizing the complex as shown in Figure 6 (a & b).

### Gene Expression Profile by RT- PCR

**Figure 7:**
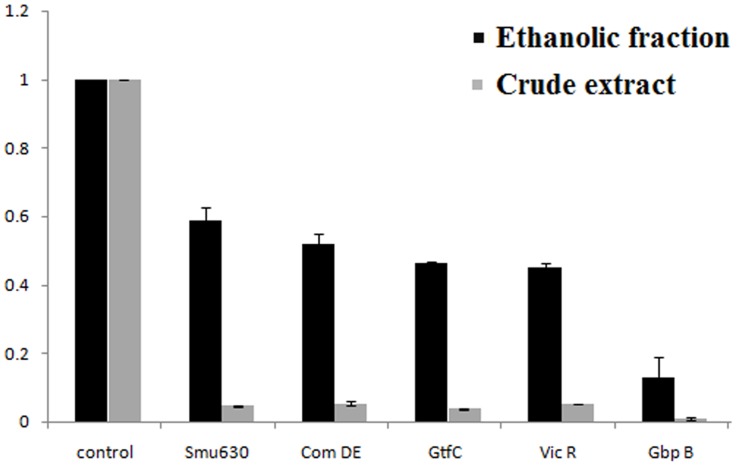
Gene expression profile of specific genes involved in the formation of biofilm qRT-PCR was carried out in triplicate. Data presented were generated from at least four independent sets of experiments (Data =  mean±SD).

The expression profile of some biofilm forming genes (smu0630, com DE, gtf C, vic R and gbp B) was determined on treated (with crude extract and ethanolic fraction) and untreated (control) biofilm cells (Figure 7). The expression data showed down regulation of these virulence genes in the treated cells. The crude extract showed extraordinary reduction in expression of the genes (up to 90%). The most affected gene was found to be gbp B, showing more than 90% reduction as compared to control. Whereas, gbp B was found suppressed by 80% in the presence of ethanolic fraction.

## Discussion

The essential role of *Streptococcus mutans* in the pathogenesis of caries is well authenticated. It still continues to affect a large section of population. It establishes the infection by influencing various virulence traits like acidogenecity, synthesis of exopolysaccharides (glucan), aciduracity, hydrophobicity, cell to cell signalling (quorum sensing), adherence and biofilm formation. Novel strategies should be developed in order to aim at the elimination of such expression of virulence. In the past, medicinal plants have provided an inspirational array of novel drug compounds and haven’t shown any sign of adverse reaction or resistance unlike antibiotics. This report, however, is first that offers insight studies and evidences that the fruit extracts of *E. officinalis* shows an outstanding cariostatic potential by preventing the virulence expression of the bacteria without suppressing its population unlike a range of chemotherapeutic strategies that prevent the growth of this oral bacteria.

The results obtained from the present study are evident that the extracts of *E.officinalis* show apparent reduction in the virulence factors of *S. mutans.* However, the crude extract consistently shows a better activity than ethanolic fraction.

The GC- MS analysis of the extracts of *E. officinalis* gave two distinctively broad peaks confirming the presence of two major constituents in each extract. The major compounds were phthalic acid and furfuraldehyde in case of crude and ethanolic fractions respectively. These compounds exhibited antimicrobial activity with MIC values of 250 µg/ml (Phthalic acid) and 500 µg/ml (furfuraldehyde) but did not affect the main virulence properties (biofilm, adherence, hydrophobicity and pH). Since our focus is not on suppressing the population of *S. mutans* in the oral cavity, rather, reducing their virulence expression. Hence, these purified compounds did not prove to be effective against cariogenic properties of bacteria.

Adherence is one of the most important step in the cascade of caries formation and its prevention could be a prophylaxis against the reduction of its virulence [24]. In glass- dependent adherence study, the glass surface was used in order to mimic the hard surface of the tooth [25]. The results obtained shows apparently that the adherence was markedly inhibited by the sub-MIC concentrations of the extracts as compared to control. The sucrose-dependent adherence was reduced to a greater extent than sucrose-independent adherence (Figure 1 a & b). Adherence is mainly due to the hydrophobic interactions between the cells and the adhering surface. The reduction in adherence could be due to the effect of flavonoids from the plant extracts which reduce the hydrophobicity of the bacteria.

The attachment of cells on a polystyrene surface casts a model for the in vitro biofilm formation of bacteria [26]. The same approach was used to study the biofilm of S. mutans in the presence of increasing concentrations of the extracts. The dye released by the bound cells is proving formation of monolayer and reduction in the number of cells adhering to the surface in the presence of the extracts. The OD at 600 nm decreases with the increase in concentration of the extracts. However the reduction in the presence of salivary pellicle was slightly more (figure 1 c & d) due to the presence of Lactoferrin, a component of saliva which is a defence factor against *Streptococcus mutans* [27].

The synthesis of extracellular glucan is an integral component of the sucrose-dependent colonization of tooth surfaces by *S. mutans*. Flavonoids are well known to have anti GTFase activity [28]. This enzyme plays a critical role in the conversion of sucrose to a sticky substance called glucan. It is well established that water-insoluble glucans synthesized from sucrose by GTFs play a vital role in mediating the adherence and colonization of *S. mutans* on the tooth surface [29]. The total proteins that comprised GTFs, secreted in the presence and absence of the plant extracts were used for in vitro synthesis and estimation of water-soluble and water- insoluble glucan. It is evident from the data that there is a prodigious reduction in the glucan formation. The inhibition of water-insoluble polysaccharide was greater when compared to water-soluble polysaccharide in this study. Since water-insoluble glucans play significant role in adhesive interactions therefore a substantial impact on the reduction of biofilm formation was observed.

*Streptococcus mutans* attaches to the tooth surface via hydrophobic interactions. The therapeutic agents which can reduce the hydrophobic bond formation can reduce the chances of caries [30]. Based on the results, the increase in concentration of extracts decreased the hydrophocity of *S. mutans* (Figure 2 c & d). Furthermore, the cell-surface hydrophobicity is said to be associated with cell-surface proteins [31]. The possible reason of this reduction in hydrophobicity could be the binding of active components of *E. officinalis* extract to the proteins associated with the cell surface.

The ability to generate acid (acidogenecity) and to function at low pH (acidurance), appear to be the main physiological factors associated with the cariogenic potential of *Streptococcus mutans* [2]. Sub-MIC concentrations of the CR and ETH extracts were found to be associated with a gradual decrease in the acid production in a dose- dependent manner. Cariostatic effect can be achieved by reducing the acid production or by inhibiting the activity of enzyme associated with the growth and glycolysing systems of *S. mutans* [32].

The growth curves (Figure 3) of the vehicle control and the samples treated with crude and ethanolic fraction gave a typical sigmoidal pattern. There was hardly any difference in the log phase of the control and the treated samples. The similarity in the pattern of the curves clearly indicates that growth of bacteria was not inhibited by the presence of extracts. These extracts, therefore, inhibited the virulence traits without affecting the bacterial viability.

Confocal Laser Scanning Microscopy images exhibit the architecture of cells in a biofilm. The cells in control are embedded in a polysaccharide matrix that accelerates cell clustering [33]. Whereas the treated samples shows dispersion proving the reduction in the glucan that stimulated clumping in case of control.

Scanning electron microscopy reveals the impact of the extracts in reducing the glucan synthesis. The cells in vehicle control are clearly embedded in a polysaccharide matrix (glucan) that stimulates cell clumping [33]. Therefore there was no dispersion seen in untreated samples. However, as supported by the results in the reduction of glucan formation by CR extract and ETH fraction, when cells are exposed to sub inhibitory concentration of these extracts (Figure 4b), the biofilm architecture is doubtlessly disturbed. There is apparent individual scattering of the cells which clearly indicates the reduction in the polysaccharide matrix which would have otherwise stimulated clumping.

ELISA of total protein to the polyclonal antibody of Ag I/II raised in rabbit were used to compare the concentration of this protein in the vehicle control and samples treated with sub inhibitory concentration of the extracts (CR and ETH). It was found that the expression of this protein was constitutively less in both the treated samples as compared to the control. It clearly indicated the reduction of surface protein antigen Ag I/ II which is known to play a crucial role in adhesion and ultimately biofilm formation [34].

Docking studies on identified compounds support the prediction of conformation and binding affinity of the molecules against a given target protein. Thus, the docking simulation against the Adhesion (Ag I/ II) was performed. This protein is known to play a crucial role in adhesion and biofilm formation [34].The range of docking score in the compounds present on crude extract (80–86) was better than those present in ethanolic fraction (76–82). These results, thus, are in agreement with our study that crude extract had shown a better activity throughout than the ethanolic fraction. Furthermore, each compound other than the major components of the extracts (phthalic acid and furfuraldehyde) gave an exceptional docking score (Ethyl stearate, 81.52; methyl palmitate, 81.45; ethyl linoleate, 80.25; hexadecane, 82.95; elaidic acid, 81.08; linoleic acid, 79.11 and octadecanoid acid, 76.09). Hence, this data suggests synergistic effect of these compounds in order to suppress Ag I/ II adhesin protein.

The levels of expression of preferred virulence genes were compared in the presence and absence of plant extracts (CR and ETH). Out of two, the crude extract proved to be more effective in reducing the expression of genes. The CR fraction perceptibly reduced the expression of *gbp B* gene when compared to the ethanolic fraction. The *gbp B* (glucan binding protein), has been recognized to play a major role in promoting plaque formation. Another gene that was suppressed to a great extent was *gtf C* (glucosyltransferases). It is responsible for water- insoluble glucan synthesis and to a lesser extent soluble glucan synthesis. Furthermore, a gene designated as *vic R* is a two component regulatory system. It is known to directly regulate a set of genes encoding for important surface proteins which are critical for adherence to a smooth tooth surface. In addition to this, gene *com DE* was also suppressed dramatically. This cell density- dependent Com system is known to regulate the genetic competence development in *S. mutans.* Therefore, the reduction in these virulence genes which are responsible for plaque cohesion leading to dental caries reflects the striking therapeutic potential of the extracts of *E. officinalis* of being cariostatic. Also, Quorum sensing system primarily comprise of the Competence Stimulating Peptide (CSP) and the two-component signal transduction system (Com DE) [35]. Hence the suppression of this gene attenuates quorum sensing mechanism resulting in despaired virulence expressions associated with it.

The results clearly indicate that the cariogenic potential shown by the plant is due to the synergistic effect of different compounds present in the plant extracts rather than the pure compounds. Hence, the extracts of *E. officinalis* consistently proved to be better than the pure compounds (phthalic acid and furfuraldehyde) in terms of being anticariogenic. These pure compounds are known to be toxic which might be the reason for lower MIC values. Furthermore, the standard anti plaque agent (cholorhexidine), which is being used in most of the dental care products, has several reports of being genotoxic [36]. On the contrary, the fruit of *Emblica officinalis* (amla) is consumed raw which rules out every possibility of being toxic.

This study concludes that *E. officinalis* extract could be a promising agent for targeting biofilms and other cariogenic properties of *S. mutans.* Hence it could be a potential antiplaque agent. Furthermore, its property of being non toxic makes it healthier to be proposed for the preparation of mouth washes and sugar free chewing gums.
